# The value of contrast-enhanced ultrasound in vascular injury from blunt abdominal trauma in solid organs: Comparison with multidetector computed tomography using angiography as the reference standard

**DOI:** 10.1097/MD.0000000000034323

**Published:** 2023-07-21

**Authors:** Jisun Lee, Yook Kim, Kyung Sik Yi, Chi-Hoon Choi, Sang-Yong Eom

**Affiliations:** a Department of Radiology, College of Medicine, Chungbuk National University, Chungbuk National University Hospital, Cheongju, Republic of Korea; b Department of Preventive Medicine, College of Medicine, Chungbuk National University, Cheongju, Republic of Korea.

**Keywords:** abdominal injuries, angiography, contrast media, multidetector computed tomography, trauma, ultrasonography, vascular injuries

## Abstract

To evaluate the accuracy of contrast-enhanced ultrasound (CEUS) for assessing vascular injury from blunt abdominal trauma in solid organs using angiography as the reference standard and to compare it with contrast-enhanced multidetector computed tomography (MDCT). Forty-nine patients with 52 blunt abdominal trauma lesions who underwent CEUS, MDCT, and angiography were enrolled in this retrospective study. Injuries included the liver (n = 23), kidney (n = 10), and spleen (n = 19). Vascular injury in solid organs was classified into 3 types: isolated pseudoaneurysm, pseudoaneurysm with low-velocity extravasation, and active bleeding. The sensitivity, specificity, positive predictive value (PPV), negative predictive value (NPV), and accuracy of CEUS and MDCT for the detection and classification of vascular injury in solid organs were calculated based on angiography. The receiver operating characteristic curve analysis of each test was performed and compared. Thirty-nine vascular injuries in solid organs were detected and classified into 9 isolated pseudoaneurysms, 9 pseudoaneurysms with low-velocity extravasation, and 21 active bleeding based on angiography as the reference standard. The sensitivity, specificity, PPV, NPV, and accuracy for bleeding detection were 97.44%, 100.00%, 100.00%, 92.86%, and 98.08%, respectively, for CEUS and MDCT. The sensitivity, specificity, PPV, NPV, and accuracy of classification (isolated pseudoaneurysm vs. pseudoaneurysm with low-velocity extravasation or active bleeding) of bleeding were 96.67%, 87.50%, 96.67%, 87.50%, and 94.74%, respectively, for CEUS and 100.00%, 75.00%, 93.75%, 100.00%, and 94.74%, respectively, for MDCT. The area under the receiver operating characteristic curves of CEUS and MDCT for bleeding detection was 0.987, and the area under the receiver operating characteristic curves for CEUS and MDCT bleeding classification were 0.921 and 0.875, respectively. CEUS and MDCT exhibited comparable consistency with angiography for detecting and classifying vascular injury from blunt abdominal trauma in solid organs. Therefore, CEUS may be an accurate and rapid imaging tool to detect bleeding and determine the need for transcatheter arterial embolization. We suggest that CEUS could be considered a first-line approach during the preparation time before MDCT to determine the appropriate management for blunt abdominal trauma.

## 1. Introduction

Active bleeding is the leading cause of death among patients with blunt abdominal trauma. The most important aspect of blunt abdominal trauma management is diagnosing hemorrhage in the solid abdominal organ and determining whether urgent intervention is necessary. As clinical examinations do not always provide sufficient information on the extent of solid organ injuries, reliable imaging techniques should be used to diagnose the disease accurately.^[1]^

Contrast-enhanced multidetector computed tomography (MDCT) is the best imaging modality in emergency assessment.^[2,3]^ However, MDCT a time-consuming, and there is a risk of further injury during transfer. In contrast, conventional abdominal ultrasound (US), also called focused assessment with sonography for trauma, can be performed directly at the bedside regardless of hemodynamic status in patients with blunt abdominal trauma. Focused assessment with sonography for trauma is a rapid, repeatable, noninvasive, and real-time examination technique with high sensitivity to detect free peritoneal fluid but low sensitivity to detect traumatic lesions in solid abdominal organs.^[4–6]^ Fortunately, with the development of new ultrasound contrast agents, contrast-enhanced ultrasound (CEUS) has improved sensitivity to detect and characterize focal lesions in solid organs compared to the existing conventional US.^[7,8]^ Many clinical studies have investigated the effectiveness of CEUS in detecting solid organ injury in blunt abdominal trauma,^[9,10]^ and a recent systematic review and meta-analysis reported that CEUS conducted in the emergency room had good accuracy in diagnosing solid organ injury compared to MDCT.^[11]^

Over the past decades, managing blunt abdominal trauma has changed from operative to nonoperative treatments, such as transcatheter arterial embolization (TAE). In particular, TAE improves the success rate of management by simultaneously diagnosing and treating active bleeding.^[12]^ When intra-abdominal bleeding occurs, the mortality rate increases by 1% every 3 minutes before bleeding is controlled.^[13]^ Therefore, an accurate and rapid imaging tool to detect bleeding is essential to determine the need for TAE.

To our knowledge, no studies have compared the efficacy of CEUS in detecting and classifying vascular injury from blunt abdominal trauma in solid organs with MDCT based on angiography. Therefore, we aimed to evaluate the accuracy of CEUS for assessing vascular injury from blunt abdominal trauma in solid organs using angiography as the reference standard and to compare it with MDCT.

## 2. Materials and methods

### 2.1. Patients

The Institutional Review Board of our institution approved this retrospective study (Institutional review board no. 2022-09-012-001), and the requirement for informed consent was waived.

A total of 54 consecutive patients with 57 blunt abdominal trauma lesions were enrolled in this retrospective study from January 2018 to December 2018. We included patients admitted to our trauma center after blunt abdominal trauma and met the trauma team activation criteria according to the American College of Surgery-Committed on Trauma.^[14]^ We have the following treatment policy for patients with blunt abdominal trauma. Patients with American Association for the Surgery of Trauma (AAST) grade III or higher solid organ injuries with/without active bleeding and those with any grade of AAST solid organ injuries with active bleeding are treated with endovascular procedures. Patients with AAST grade I or II solid organ injuries without active bleeding are treated with conservative therapies. The inclusion criteria of this study were: patients with AAST grade III or higher solid organ injuries with or without active bleeding depicted on MDCT and CEUS and requiring angiography; and patients with AAST grade I or II solid organ injuries without active bleeding on MDCT and CEUS but underwent angiography for clinical reassurance. Of the 54 patients, 3 patients with inadequate CEUS imaging for analysis due to poor sonic window and 2 patients with AAST grade I or II solid organ injuries without active bleeding on both MDCT and CEUS but without follow-up until full recovery were excluded from the analysis. Finally, 49 consecutive patients (32 males, 17 females; age range, 20–85 years; median age 44.5 years; interquartile range, 32–62.5 years) with 52 lesions (23 liver, 19 spleen, and 10 kidney injuries) were included in this study, as presented in Table 1. Motor vehicle collisions and accidental falls were the most frequent causes of injury, followed by automobile–pedestrian collisions, motorcycle collisions, and crush injuries. The median injury severity score at presentation was 23.5 (range, 13–43; interquartile range, 19–26.5).^[15]^

**Table 1:** Baseline characteristics of the patients.

Characteristics	Number of patients (n = 49)	Percent
Age*	44.5 (32–62.5)	–
Gender		
Men	32	65.30
Women	17	34.70
Injured organ (n = 52)		
Liver	23	44.23
Spleen	19	36.53
Kidney	10	19.23
Injury mechanism		
Motor vehicle collision	13	26.53
Fall	13	26.53
Auto-pedestrian collision	9	18.37
Motorcycle collision	8	16.33
Crush injury	6	12.24
ISS at presentation*	23.5 (19–26.5)	–

ISS = Injury severity score.

* Data are presented as median (interquartile range).

### 2.2. Contrast-enhanced ultrasound

CEUS was performed using a second-generation blood pool contrast agent (Sono Vue; Bracco, Milan, Italy). A 2.4 mL Sono Vue bolus was administered through an antecubital vein, followed by a 5–10 mL normal saline flush. CEUS examination was performed with a US device (Logiq P9; GE Healthcare, Chicago, IL) using a curved transducer (C1-5-RS; bandwidth: 1–6 MHz) at a low mechanical index (0.14–0.16). The patients underwent CEUS examination at the bedside in the emergency room just before or within 1 hour after MDCT. All CEUS examinations were performed by one of two experienced radiologists with at least 5 years of experience in emergency radiology and trauma imaging. As a routine procedure, in sequence, an initial CEUS examination of the kidney, liver, and spleen was completed for each patient in 5 to 8 minutes. A second CEUS examination was performed, focusing on the abnormality detected on the first examination with continuous image acquisition, starting immediately after contrast injection and lasting 5 minutes.

### 2.3. Contrast-enhanced MDCT

All MDCT scans were performed on a 256-detector computed tomography (CT) scanner (Revolution CT; GE Healthcare, Waukesha, WI) within 2 hours of the emergency department visit. A volume of 1.5 mL/kg body weight of nonionic contrast medium (Xenetics 350, Guerbet, Roissy, France; Omnipaque 350, GE Healthcare; Iomeron 350, Bracco, Milan, Italy) was injected at a rate of 3 to 3.5 mL/s using a power injector. The acquisition of arterial phase images was set to begin after 12 seconds when a specific circular region in the abdominal aorta at the vertebral body L3 level reached 150 Hounsfield units, as determined using the bolus tracking method. Portal venous and delayed phase images were acquired 70 to 80 and 180 seconds, respectively, after injecting the contrast medium.

### 2.4. Angiography

Angiography and TAE were performed by a radiologist (6 years of experience in interventional radiology) using an angiographic device with a flat-panel detector of a 2480 × 1920 element (Allura Xper FD20; Philips Healthcare, Best, the Netherlands). An initial anteroposterior hepatic, splenic, or renal angiogram was performed according to the target organs suspected to have been injured on previous imaging examinations. For patients with active bleeding on angiography, TAE was performed after selecting the target vessel using a microcatheter and embolic agents, including absorbable gelatin sponge, micro coils, and N-butyl cyanoacrylate.

### 2.5. Image analysis

The CEUS and MDCT images were retrospectively analyzed by 2 radiologists (J.L. and Y.K. with more than 8 years of experience in abdominal imaging) who were not involved with the original clinical examination of the patients’ studies. They were unaware of the clinical data of the patients, the findings of CEUS or MDCT, and angiography results. In case of disagreement, the reviewers reached a consensus by jointly reviewing the images with a third radiologist (B.S.C. with 12 years of experience in abdominal imaging).

A positive finding indicating vascular injury in the solid organ on CEUS, MDCT, and angiography was defined as the presence of an extravasated contrast agent. In this study, we classified vascular injury in solid organs into 3 types: isolated pseudoaneurysm, pseudoaneurysm with low-velocity extravasation, and active bleeding. An isolated pseudoaneurysm was depicted as a contrast pooling represented by a hyperechoic, round, or oval spot on CEUS, a well-defined round or oval hyperdense lesion on MDCT, and a well-defined round or oval structure that was enhanced from the parent artery on angiography in the arterial phase without change of shape in the portal phase. Pseudoaneurysm with low-velocity extravasation was described as a contrast pooling represented by a hyperechoic round or oval lesion with a dot sign or short-tail sign on CEUS, well-defined round or oval hyperdense lesion with contrast blush on MDCT in the arterial and portal phases, and a well-defined round or oval structure that enhances from the parent artery with minimal change of shape or residual sign of leaked contrast agent after the initial angiography. Active bleeding was defined as a jet of opacified blood represented by a fountain-like hyperechoic lesion with a long-tail sign on CEUS, an ill-defined linear or layering jet-like hyperdense lesion on MDCT, and a jet of contrast agent arising from the parent artery on angiography in the arterial phase with an increase in extent in the portal phase.

### 2.6. Statistical analysis

Continuous variables, including age and injury severity score at presentation were tested by a 2-sample *t* test. Categorical variables including sex, injured organ, and injury mechanism were tested by the chi-square test.

Sensitivity, specificity, positive predictive value (PPV), negative predictive value (NPV), and accuracy of CEUS for the detection and classification of vascular injury in solid organs were calculated using angiography as the standard reference and were compared with the results of MDCT. For detection evaluation, the 2 groups were divided into positive and negative findings for bleeding in each examination. In classification evaluation, positive findings indicating vascular injury were divided into 2 groups (isolated pseudoaneurysm vs. pseudoaneurysm with low-velocity extravasation or active bleeding) for each examination. The McNemar test was used to compare the diagnostic performances of CEUS and MDCT.

Receiver operating characteristic curve analysis was performed for each test. The area under the receiver operating characteristic curve (AUC) was calculated separately for CEUS and computed tomography to detect and classify bleeding.

The kappa statistic was used to assess the inter-reader agreement for detecting and classifying bleeding on CEUS. Kappa value indicated poor agreement (κ < 0.20); fair agreement (κ = 0.21–0.40); moderate agreement (κ = 0.41–0.60); good agreement (κ = 0.61–0.80); and excellent agreement (κ = 0.81–1.00).^[16]^

All statistical analyses were performed using MedCalc for Microsoft Windows version 18 (MedCalc Software, Mariakerke, Belgium). A *P* value less than .05 was considered statistically significant.

## 3. Results

### 3.1. Results of vascular injury in solid organ from blunt abdominal trauma identified on CEUS, MDCT, and angiography

**Figure 1.**
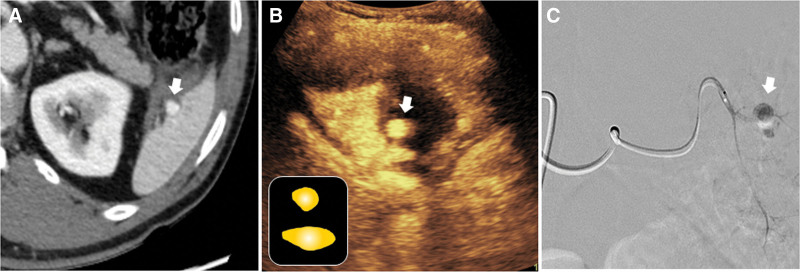
Isolated pseudoaneurysm of the spleen in a 16-year-old-female after falling. (A) Arterial phase of the contrast-enhanced axial image CT scan demonstrate a contained vascular injury (arrow) suggesting pseudoaneurysm in the spleen. (B) Subsequent CEUS image reveals a round-shaped contrast pooling (arrow) detected on the CT scan. Schematic illustration of vascular injury in solid organ in the CEUS indicating isolated pseudoaneurysm is in the small box at the bottom left. (C) Selective angiogram of the splenic artery by microcatheter reveals a pseudoaneurysm (arrow) that corroborates the initial CT and CEUS findings. CEUS = contrast-enhanced ultrasound, CT = computed tomography.

**Figure 2.**
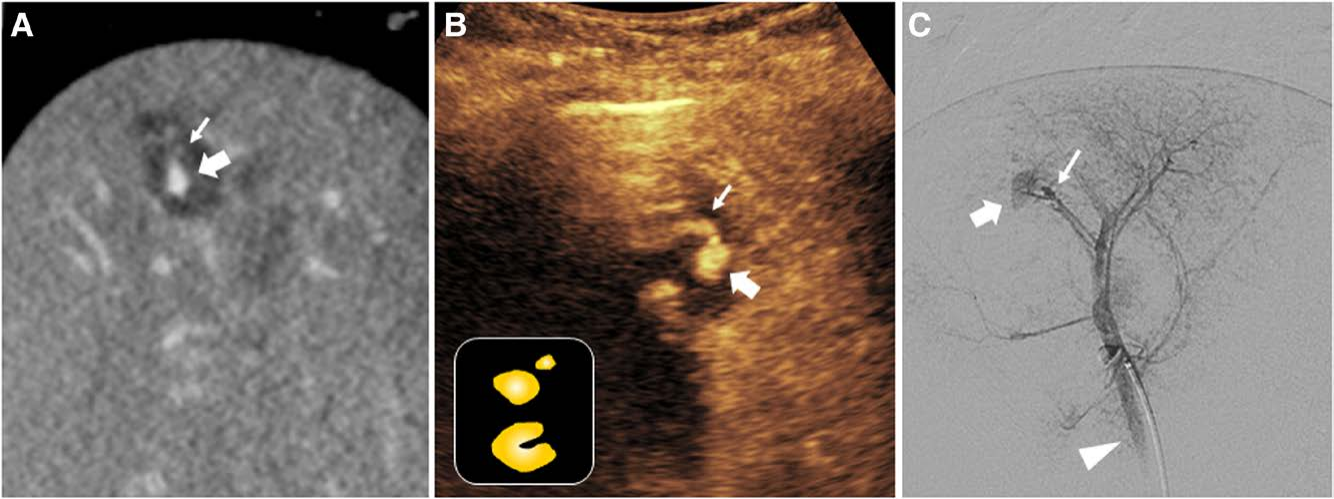
Pseudoaneurysm with low-velocity extravasation with parenchymal hematoma of the liver in a 50-year-old-female after falling. (A) Contrast-enhanced coronal CT scan reveals parenchymal hematoma with a well-defined oval hyperdense lesion (thick arrow) and contrast blush (thin arrow), suggesting pseudoaneurysm with low-velocity extravasation in the liver. (B) CEUS image reveals small contrast pooling (thick arrow) with adjacent short tail signs (thin arrow) that are observed in the initial CT scan. A schematic illustration of vascular injury in a solid organ in the CEUS indicating pseudoaneurysm with low-velocity extravasation is in the small box at the bottom left. (C) The selective angiogram of the hepatic artery confirms the pseudoaneurysm (thick arrow) from the branch of the hepatic artery with focal contrast media extravasation (thin arrow) and early drainage of the hepatic vein suggesting arteriovenous fistula (arrowhead). CEUS = contrast-enhanced ultrasound, CT = computed tomography.

**Figure 3.**
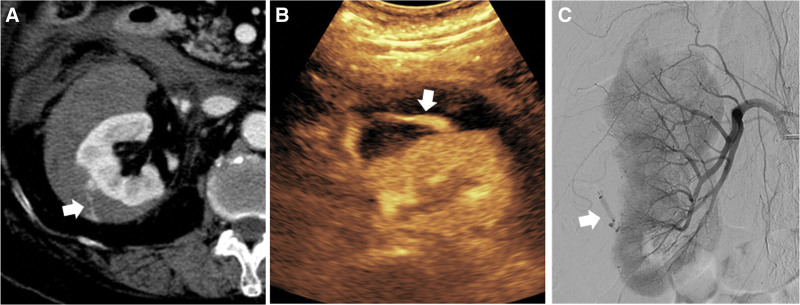
Renal parenchymal laceration with active bleeding in an 86-year-old female after a motorcycle accident. (A) Arterial phase contrast-enhanced CT scan reveals extravasation of contrast media at perirenal space (arrow). (B) The CEUS image demonstrates a fountain-like hyperechoic lesion with a long-tail sign (arrow) from the kidney, suggesting active bleeding. A schematic illustration of vascular injury in solid organ in the CEUS indicating active bleeding is in the small box at the bottom left. (C) The angiogram of the renal artery is performed, confirming active bleeding from the renal artery (arrow). CEUS = contrast-enhanced ultrasound, CT = computed tomography.

Based on angiography, which was the reference standard, we analyzed the accuracy of CEUS and MDCT to identify vascular injury from blunt abdominal trauma in solid organs. Thirteen out of 52 lesions (25.00%) were confirmed as negative bleeding, and 39 (75.00%) were positive and were classified into 9 isolated pseudoaneurysms (17.31%; Fig. 1), 9 pseudoaneurysms with low-velocity extravasation (17.31%; Fig. 2), and 21 active bleeding events (40.38%; Fig. 3).

According to the CEUS results, 14 out of 52 lesions (26.92%) had negative bleeding, 38 lesions (73.08%) had positive bleeding, 8 isolated pseudoaneurysms (15.38%), 11 pseudoaneurysms with low-velocity extravasation (21.15%), and 19 active bleeding (36.54%). One of the 14 negative bleeding findings detected on CEUS was recognized as an isolated pseudoaneurysm on angiography. One of eight isolated pseudoaneurysms depicted on CEUS was recognized as active bleeding on angiography. Two of the 11 pseudoaneurysms with low-velocity extravasation on CEUS were misdiagnosed and confirmed as isolated pseudoaneurysms and active bleeding on angiography, respectively. All 19 active extravasations detected on CEUS were identical to those on the angiography results.

**Figure 4.**
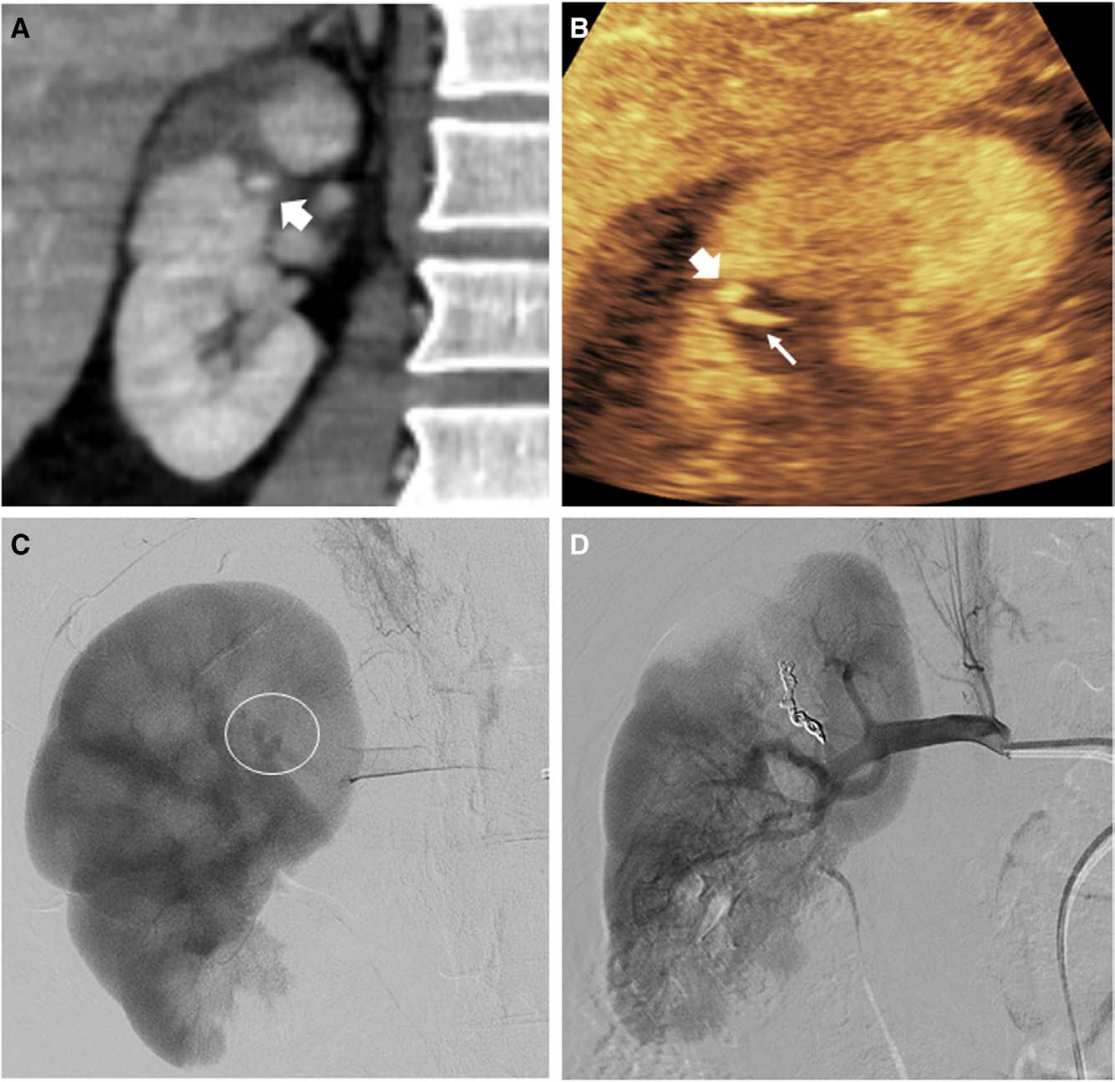
Segmental kidney infarction with combined vascular injury in a 46-year-old male after falling. (A) CT scan reveals segmental renal infarction with internal focal high-density foci suggesting pseudoaneurysm (arrow). An isolated pseudoaneurysm detected on CT may be observed for an increase in size as a conservative treatment. (B) Subsequent CEUS reveals a hyperechoic oval lesion with a short-tail sign suggesting pseudoaneurysm with low-velocity extravasation (thin arrow) requiring further evaluation, such as angioembolization. (C) The delayed image of the renal artery angiogram revealing a leaked contrast agent indicates that this is not an isolated pseudoaneurysm, which is consistent with the CEUS findings. (D) Complete total embolization of the renal artery was performed using coil and gel foam, and a subsequent angiography revealed successful complete obliteration of the renal artery. CEUS = contrast-enhanced ultrasound, CT = computed tomography.

**Figure 5.**
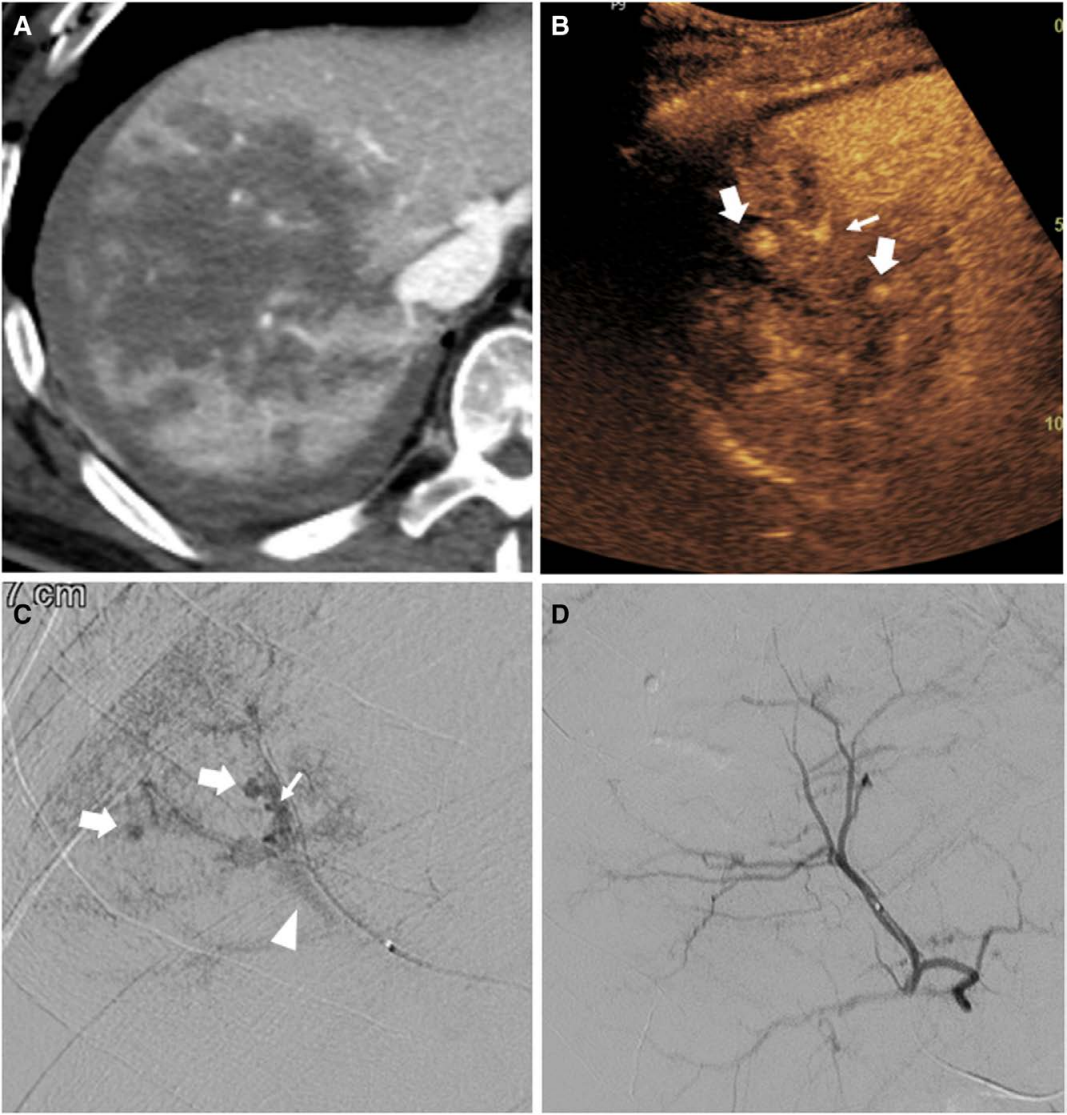
Liver capsular vascular injury combined with bare area hematoma in a 73-year-old male after a motor vehicle accident. (A) Contrast-enhanced axial CT scan reveals a large non-enhancing lesion in the right liver and multiple enhancing foci; however, it is unclear whether it is vascular injury requiring angioembolization or partial volume artifacts of normal vascular structure. (B) The subsequent CEUS demonstrates the vascular injury as hyperechoic round spots (thick arrow) and fountain-like hyperechoic lesions with long-tail signs (thin arrow), suggesting pseudoaneurysms and active bleeding. (C) Given the CEUS finding, selective angiogram of the right hepatic artery is performed, which demonstrates multiple pseudoaneurysms (thick arrow) and active bleeding (thin arrow), and early drainage of the hepatic vein suggesting arteriovenous fistula (arrowhead). (D) Complete total embolization of the right hepatic artery was performed using glue, and a subsequent angiography revealed successful complete obliteration of the right hepatic artery. In this case, CEUS could accurately diagnose the site of trauma-related vascular injury and guide the subsequent embolization procedure. CEUS = contrast-enhanced ultrasound, CT = computed tomography.

On MDCT, 14 out of 52 lesions (26.92%) had negative bleeding, 38 lesions (73.08%) had positive bleeding, 8 isolated pseudoaneurysms (15.38%), 8 pseudoaneurysms with low-velocity extravasation (15.38%), and 22 had active bleeding (42.31%). One of the 14 negative bleeding detected on MDCT was confirmed as an isolated pseudoaneurysm on angiography. Two out of eight isolated pseudoaneurysms detected on MDCT were revealed to be pseudoaneurysms with low-velocity extravasation on angiography (Fig. 4). Three of eight pseudoaneurysms with low-velocity extravasation depicted on MDCT were revealed to be active extravasations on angiography (Fig. 5). Four out of 22 active bleedings on MDCT were misdiagnosed and confirmed as 2 isolated pseudoaneurysms and 2 pseudoaneurysms with low-velocity extravasation on angiography. Inter-reader agreement on bleeding classification in CEUS and MDCT results assessed by Kappa value was excellent (κ = 0.91 and κ = 0.93, respectively).

In our study, there were no contraindications for TAE, in which liver necrosis was a concern; therefore, we actively treated all vascular injuries.

### 3.2. Assessment of vascular injury in solid organ from blunt abdominal trauma identified on CEUS and MDCT with angiography as the reference standard

Thirteen lesions confirmed as negative bleeding on angiography revealed the same results on CEUS and MDCT. Among 39 lesions with positive bleeding on angiography, 38 lesions (97.44%) in CEUS and MDCT revealed the same results. Of the 9 confirmed isolated pseudoaneurysms in angiography, 7 lesions (77.78%) revealed the same result in CEUS and 6 lesions (66.67%) in MDCT; of the 9 confirmed pseudoaneurysms with low-velocity extravasation in angiography, all 9 lesions (100.00%) revealed the same result in CEUS, and 7 lesions (77.78%) revealed the same result in MDCT. There were 21 lesions confirmed as active extravasations in angiography, of which 19 lesions (90.48%) revealed the same result in CEUS and 18 lesions (85.72%) in MDCT (Table 2).

**Table 2:** Assessment of vascular injury in solid organs from blunt abdominal trauma identified on CEUS and MDCT with angiography as a reference standard.

Assessment of bleeding in solid organ injury		Number of cases on angiography	Number of cases detected/total number of cases (%)	
			CEUS	MDCT
Detection	Negative bleeding	13	13/13 (100)	13/13 (100)
	Positive bleeding	39	38/39 (97.44)	38/39 (97.44)
Classification	Isolated pseudoaneurysm	9	7/9 (77.78)	6/9 (66.67)
	Pseudoaneurysm with low-velocity extravasation	9	9/9 (100)	5/9 (55.56)
	Active extravasation	21	19/21 (90.48)	18/21 (85.71)

CEUS = contrast-enhanced ultrasound, MDCT = multidetector computed tomography.

### 3.3. Diagnostic performance for detection and classification of vascular injury from blunt abdominal trauma in solid organ using angiography as the reference standard

The sensitivity, specificity, PPV, NPV, and accuracy for detecting bleeding were 97.44%, 100.00%, 100.00%, 92.86%, and 98.08%, respectively, for CEUS and MDCT (Table 3). The sensitivity, specificity, PPV, NPV, and accuracy of classification (isolated pseudoaneurysm vs. pseudoaneurysm with low-velocity extravasation or active extravasation) of bleeding were 96.67%, 87.50%, 96.67%, 87.50%, and 94.74%, respectively, for CEUS and 93.33%, 75.00%, 93.33%, 75.00%, and 89.47%, respectively, for MDCT (Table 4).

**Table 3:** Diagnostic performance for detecting vascular injury from blunt abdominal trauma in solid organs using angiography as the reference standard.

	Sensitivity	Specificity	Positive predictive value	Negative predictive value	Accuracy
CEUS	97.44%	100.00%	100.00%	92.86%	98.08%
MDCT	97.44%	100.00%	100.00%	92.86%	98.08%

CEUS = contrast-enhanced ultrasound, MDCT = multidetector computed tomography.

**Table 4:** Diagnostic performance for classification of vascular injury in solid organ from blunt abdominal trauma (isolated pseudoaneurysm vs pseudoaneurysm with low velocity extravasation or active bleeding) using angiography as the reference standard.

	Sensitivity	Specificity	Positive predictive value	Negative predictive value	Accuracy
CEUS	96.67%	87.50%	96.67%	87.50%	94.74%
MDCT	93.33%	75.00%	93.33%	75.00%	89.47%

CEUS = contrast-enhanced ultrasound, MDCT = multidetector computed tomography.

The AUCs of CEUS and MDCT for bleeding detection was 0.987, and the AUCs for CEUS and MDCT for bleeding classification were 0.921 and 0.842, respectively, and there was no statistically significant difference (*P* = .46).

## 4. Discussion

CEUS, a radiation-free and easily accessible technique, is a promising imaging modality for assessing traumatic abdominal lesions. Recent studies have revealed that CEUS is excellent for detecting and grading traumatic abdominal lesions with sensitivity and specificity levels of up to 95%, similar to MDCT.^[11,17,18]^

Whereas previous studies only compared the diagnostic performance of CEUS for traumatic abdominal lesions with MDCT, we used angiography as a standard reference and compared the diagnostic performance of CEUS for assessing vascular injury in solid organs from blunt abdominal trauma with that of MDCT. In this study, the bleeding detection accuracy was > 98% in CEUS and MDCT, which is consistent with previous results.^[11]^

TAE is useful for identifying vascular injury and treating bleeding concurrently; thus, it is considered a first-line treatment in patients with vascular injury in solid organs from blunt abdominal trauma. Therefore, we used angiography findings as the standard reference for assessing vascular injury in solid organs from blunt abdominal trauma on CEUS and compared them with MDCT findings. In 2019, Tagliati et al^[19]^ compared the diagnostic performance of CEUS and angiography for delayed splenic injury complications in a limited number of patients in the study group, and CEUS revealed a sensitivity of 100% and a positive predictive value of 92.3%.

The advantage of using angiography as a standard reference is that, unlike the static images of MDCT, CEUS and angiography allow real-time motion evaluation of bleeding patterns; therefore, a more detailed analysis is possible. In this study, we classified vascular injury in solid organs into 3 types based on common findings from each imaging modality: isolated pseudoaneurysm, pseudoaneurysm with low-velocity extravasation, and active bleeding. The concept of pseudoaneurysm with low-velocity extravasation is our newly proposed type of vascular injury, which appears as a simple pseudoaneurysm; however, microhemorrhage can be observed, especially as a short tail sign on CEUS. The diagnostic performance for classifying vascular injury in solid organs from blunt abdominal trauma (isolated pseudoaneurysm vs. pseudoaneurysm with low-velocity extravasation or active bleeding) using angiography as the reference standard was calculated, and the sensitivity for classification in CEUS revealed a higher tendency than in MDCT.

TAE for controlling hemorrhage in blunt abdominal trauma has been established as a safe and effective treatment; however, some patients require open surgery or conservative management.^[20]^ It is important to assess the bleeding pattern or the patient’s condition to decide whether to perform TAE. TAE should be actively performed for patients with signs of ongoing bleeding (active bleeding or pseudoaneurysm with low-velocity extravasation in our study), except for contraindications in liver necrosis or an isolated pseudoaneurysm with small size (<10 mm) and specific location, where it may thrombose spontaneously.^[21,22]^ In our study, there were no contraindications for TAE; thus we actively treated all vascular injuries. However, the detailed bleeding patterns revealed in CEUS in our study may help determine the indication for TAE.

CEUS is a promising imaging modality for various uses with many advantages; however, it has some limitations. CEUS is useful for evaluating solid abdominal organs; however, it is unsuitable for bowel examination due to the limitations from inherent permeability of ultrasound and artifacts. Owing to the characteristic metabolic process of ultrasound contrast medium, renal pelvicalyceal system or bile duct injuries cannot be evaluated using CEUS. Therefore, CEUS is appropriate as a complementary test rather than a test that replaces MDCT, which is already recognized as a primary imaging method used for total-body evaluation in blunt trauma patients. Taking advantage of the unique features of CEUS may help maximize its effectiveness.

This study had some limitations. First, it is a retrospective single-institute analysis, and the possibility of a selection bias cannot be excluded. Second, there is an inevitable difference in the time taken to perform each examination because 1 patient cannot undergo multiple tests simultaneously. Third, due to the small sample size, we could not analyze the subgroups by organ, which may have had differences. Since the patients’ prognosis according to the detailed pattern presented by our study has not been analyzed, a large-scale prospective study that excludes these limitations is needed.

## 5. Conclusions

In conclusion, CEUS and MDCT exhibited comparable consistency with angiography for detecting and classifying vascular injury from blunt abdominal trauma in solid organs. Therefore, CEUS may be an accurate and rapid imaging tool to detect bleeding and determine the need for TAE. We suggest that CEUS be considered a first-line approach during the preparation time prior to MDCT to determine the appropriate management for blunt abdominal trauma.

## Author contributions

**Conceptualization:** Jisun Lee, Yook Kim.

**Data curation:** Jisun Lee, Yook Kim, Sang-Yong Eom.

**Investigation:** Jisun Lee, Yook Kim.

**Resources:** Kyung Sik Yi, Chi-Hoon Choi.

**Supervision:** Jisun Lee, Yook Kim.

**Visualization:** Jisun Lee, Yook Kim, Kyung Sik Yi, Chi-Hoon Choi.

**Writing – original draft:** Jisun Lee, Yook Kim.

**Writing – review & editing:** Jisun Lee, Yook Kim.

## References

[1] van Beeck EF van Roijen L Mackenbach JP. Medical costs and economic production losses due to injuries in the Netherlands. J Trauma. 1997;42:1116–23. 9210552 10.1097/00005373-199706000-00023

[2] Poletti PA Wintermark M Schnyder P. Traumatic injuries: role of imaging in the management of the polytrauma victim (conservative expectation). Eur Radiol. 2002;12:969–78. 11976841 10.1007/s00330-002-1353-y

[3] Yoon W Jeong YY Kim JK. CT in blunt liver trauma. Radiographics. 2005;25:87–104. 15653589 10.1148/rg.251045079

[4] Valentino M Serra C Zironi G. Blunt abdominal trauma: emergency contrast-enhanced sonography for detection of solid organ injuries. AJR Am J Roentgenol. 2006;186:1361–7. 16632732 10.2214/AJR.05.0027

[5] Markowitz JE Hwang JQ Moore CL. Development and validation of a web-based assessment tool for the extended focused assessment with sonography in trauma examination. J Ultrasound Med. 2011;30:371–5. 21357559 10.7863/jum.2011.30.3.371

[6] Brun PM Bessereau J Chenaitia H. Stay and play eFAST or scoop and run eFAST? that is the question. Am J Emerg Med. 2014;32:166–70. 24332906 10.1016/j.ajem.2013.11.008

[7] Nicolau C Ripollés T. Contrast-enhanced ultrasound in abdominal imaging. Abdom Imaging. 2012;37:1–19. 21879317 10.1007/s00261-011-9796-8

[8] Cagini L Gravante S Malaspina CM. Contrast enhanced ultrasound (CEUS) in blunt abdominal trauma. Crit Ultrasound J. 2013;5(Suppl 1):S9. 23902930 10.1186/2036-7902-5-S1-S9 PMC3711741

[9] Afaq A Harvey C Aldin Z. Contrast-enhanced ultrasound in abdominal trauma. Eur J Emerg Med. 2012;19:140–5. 21934507 10.1097/MEJ.0b013e328348c980

[10] Pinto F Miele V Scaglione M. The use of contrast-enhanced ultrasound in blunt abdominal trauma: advantages and limitations. Acta Radiol. 2014;55:776–84. 24060814 10.1177/0284185113505517

[11] Zhang Z Hong Y Liu N. Diagnostic accuracy of contrast enhanced ultrasound in patients with blunt abdominal trauma presenting to the emergency department: a systematic review and meta-analysis. Sci Rep. 2017;7:4446. 28667280 10.1038/s41598-017-04779-2 PMC5493732

[12] Salcedo ES Brown IE Corwin MT. Angioembolization for solid organ injury: a brief review. Int J Surg. 2016;33(Pt B):225–30. 26537314 10.1016/j.ijsu.2015.10.030

[13] Clarke JR Trooskin SZ Doshi PJ. Time to laparotomy for intra-abdominal bleeding from trauma does affect survival for delays up to 90 minutes. J Trauma. 2002;52:420–5. 11901314 10.1097/00005373-200203000-00002

[14] Colonna AL Meredith JW. American College of Surgeons Resources Document. In: Vincent J-L Hall JB, eds. Encyclopedia of Intensive Care Medicine. Berlin Heidelberg: Springer; 2012:155–60.

[15] Baker SP O’Neill B Haddon W. The injury severity score: a method for describing patients with multiple injuries and evaluating emergency care. J Trauma. 1974;14:187–96. 4814394

[16] Cohen J. Weighted kappa: nominal scale agreement with provision for scaled disagreement or partial credit. Psychol Bull. 1968;70:213–20. 19673146 10.1037/h0026256

[17] Dormagen J Meyerdierks O Gaarder C. Contrast-enhanced ultrasound of the injured spleen after embolization – comparison with computed tomography. Ultraschall Med. 2011;32:485–91. 21294071 10.1055/s-0029-1246003

[18] Sessa B Trinci M Ianniello S. Blunt abdominal trauma: role of contrast-enhanced ultrasound (CEUS) in the detection and staging of abdominal traumatic lesions compared to US and CE-MDCT. Radiol Med. 2015;120:180–9. 24961341 10.1007/s11547-014-0425-9

[19] Tagliati C Argalia G Polonara G. Contrast-enhanced ultrasound in delayed splenic vascular injury and active extravasation diagnosis. Radiol Med. 2019;124:170–5. 30488252 10.1007/s11547-018-0961-9

[20] Ierardi AM Duka E Lucchina N. The role of interventional radiology in abdominopelvic trauma. Br J Radiol. 2016;89:20150866. 26642310 10.1259/bjr.20150866 PMC4985465

[21] Kittaka H Yagi Y Zushi R. The investigation of posttraumatic pseudoaneurysms in patients treated with nonoperative management for blunt abdominal solid organ injuries. PLoS One. 2015;10:e0121078. 25781957 10.1371/journal.pone.0121078 PMC4363468

[22] Kagoura M Monden K Sadamori H. Outcomes and management of delayed complication after severe blunt liver injury. BMC Surg. 2022;22:241. 35733106 10.1186/s12893-022-01691-z PMC9219165

